# Influence of Surface Tension on Dynamic Characteristics of Single Bubble in Free-Field Exposed to Ultrasound

**DOI:** 10.3390/mi13050782

**Published:** 2022-05-17

**Authors:** Hao Wu, Tianshu Zhang, Xiaochen Lai, Haixia Yu, Dachao Li, Hao Zheng, Hui Chen, Claus-Dieter Ohl, Yuanyuan Li

**Affiliations:** 1 Innovative Institute of Chinese Medicine and Pharmacy, Shandong University of Traditional Chinese Medicine, Jinan 250355, China; haowu_hill@163.com (H.W.); zhangtianshu2021@163.com (T.Z.); shenhui@tmu.edu.cn (H.C.); 2 School of Automation, Nanjing University of Information Science and Technology, Nanjing 210044, China; xchlai@nuist.edu.cn; 3 State Key Laboratory of Precision Measuring Technology and Instruments, Tianjin University, Tianjin 300072, China; hxy208@tju.edu.cn (H.Y.); dchli@tju.edu.cn (D.L.); zhenghao_0402@tju.edu.cn (H.Z.); 4 Department of Soft Matter, Institute of Physics, Otto-von-Guericke University, 39106 Magdeburg, Germany; claus_dieter.ohl@ovgu.de

**Keywords:** acoustic cavitation, single bubble, surface tension, bubble dynamics

## Abstract

The motion of bubbles in an ultrasonic field is a fundamental physical mechanism in most applications of acoustic cavitation. In these applications, surface-active solutes, which could lower the surface tension of the liquid, are always utilized to improve efficiency by reducing the cavitation threshold. This paper examines the influence of liquids’ surface tension on single micro-bubbles motion in an ultrasonic field. A novel experimental system based on high-speed photography has been designed to investigate the temporary evolution of a single bubble in the free-field exposed to a 20.43 kHz ultrasound in liquids with different surface tensions. In addition, the R-P equations in the liquid with different surface tension are solved. It is found that the influences of the surface tension on the bubble dynamics are obvious, which reflect on the changes in the maximum size and speed of the bubble margin during bubble oscillating, as well as the weaker stability of the bubble in the liquid with low surface tension, especially for the oscillating bubble with higher speed. These effects of the surface tension on the bubble dynamics can explain the mechanism of surfactants for promoting acoustic cavitation in numerous application fields.

## 1. Introduction

Cavitation is a vital hydrodynamic phenomenon in many scientific and engineering fields [1,2,3]. Its advantages in applications have drawn much attention from researchers, such as its high efficiency and micro-scaled size. However, sometimes cavitation is undesirable because it causes substantial corrosion of the spinning blades, as well as additional noise from knocking and vibrations, as well as a major loss of productivity due to flow pattern distortion [4,5,6,7]. According to the means of generation, cavitation can be categorized into four types: acoustic, hydrodynamic, optic, and particle cavitations, which are all applied in various fields. Among these, ultrasonic cavitation is one of the most important cavitation patterns, which has been widely used in daily life and the industry, such as ultrasonic cleaning [8], and food or medicine processing [1,3].

In the presence of ultrasound, bubbles would oscillate, move, collapse and fuse, which is defined as acoustic cavitation and plays a fundamental role in multiple applications [8,9,10,11,12,13]. In most of these applications, surface-active solutes are usually used for the improvement of efficiency by lowering the surface tension of the liquid. It has been pointed out by researchers that more cavities and lower cavitation thresholds would be the main reasons of these improvements in most applications [14,15,16].

Camerotto et al. [17] conducted a study on the effects of lower bulk surface tension on bubble activity and surface cleaning performance. It was demonstrated that cavitation activity, measured by means of ultra-harmonic cavitation noise, is enhanced in the presence of lower surface tension. Furthermore, cavitation noise measurements were correlated to cleaning performance by means of particle removal and damage tests on patterned wafers. As a result, a clear increase in particle removal efficiency of 78 nm SiO2 particles was obtained in the liquid with lower surface tension. Curtiss et al. [18] modeled a tissue layer as a density interface acted upon by surface tension to mimic membrane effects and focused on ultrasound contrast agent type bubbles which have immediate biomedical applications, such as the delivery of drugs and the instigation of sonoporation. The effect of interfacial tension on the interaction between a single bubble and the tissue layer was explored, and an obvious increase was found in the liquid with lower surface tension. Later, some researchers found that the influence of surface tension on the dynamics of cavitation bubbles was another important factor to improve the efficiency of applications. Luo et al. [19] studied the mesoscale causes of material erosion in solutions with different surface tensions with cavitation bubbles generated by underwater low-voltage discharge technology and found that the radial velocity of cavitation bubbles decreases significantly during the shrinkage of the cavitation bubble and the minimum radius to which the cavitation bubble shrink increases as the liquid surface tension decreases, the micro-jet generated by the cavitation bubble develops more slowly, and the impact strength of cavitation bubble collapses on the wall decreases in the liquids with lower surface tension. Sabzeghabae et al. [20] investigated the dynamics of bubbles in liquids with different surface tensions by simultaneous high-speed shadowgraph and spatial transmittance modulation. It was found that there was more efficient cavitation by laser-induced bubbles in liquids with lower surface tension and there was a direct correlation between the bubbles’ dynamics and cavitation intensity.

To reveal the underlying mechanism of acoustic cavitation, it is vital to investigate the single acoustic cavitation bubble. A large amount of research has been focused on the microscale mechanism of how the cavitation bubble is affected by the liquid surface tension. Lee et al. [21] examined the effect of surface-active solutes on the behaviors of acoustic bubbles, generated in a micro-space using a low-frequency ultrasound (60 kHz). In the experiments, the dynamic behaviors of bubbles, such as bubble coalescence, clustering, and fragmentation, could be observed directly by confining cavitation within a micro-space. It was clearly observed that the dynamics of individual bubbles were much different in the liquids with different surface tension which could be the potential effect mechanism of surface tension on improving application efficiency. Liu et al. [22,23] tried to reveal the influence of liquid surface tension on a single bubble’s dynamics near a rigid wall via the laser-induced bubbles. It was also observed that the surface-tension forces hindered the bubble growth progress, so increasing surface tension decreased the maximum bubble radii. On the other hand, the surface-tension forces speed up the bubble collapse process. In larger surface tension cases, the bubble velocity was higher, and higher erosive power is produced. Recently, Wu et al. [24] successfully captured the temporary evolution of single acoustic cavitation near a rigid wall in liquids with different surface tension with a synchronous high-speed microscopic imaging method. The results showed that the liquid surface tension not only affected the bubble’s stability in the ultrasonic field but also obviously influenced the characteristics of the bubble’s typical motions near a rigid wall, such as moving, collapse, and rebound. In addition, more researchers were more willing to analyze the influence of liquid surface tension with various numerical methods [15,16,17,18,19,20,21,22,23,24,25,26,27]. Among these methods, the R–P equation (Rayleigh–Plesset equation) is regarded as the most classic one [28].

Plenty of investigations have been made on the influence of liquid surface tension on cavitation by experiments and simulations, but there is no clear explanation of the effect mechanisms up to now. In the present work, a single bubble in free-field exposed to a low-frequency ultrasound, which is the basic model unit in most applications of acoustic cavitation, was observed and analyzed in liquids with different surface tensions. Compared to our previous work, we focus more on the tiny influence of liquid’s surface tension on bubble’s oscillation, and the relationship between the influence of liquid’s surface tension and the intensity of the bubble’s motion. Important results about the differences in bubble dynamics were recorded, which were compared with theoretical analyses based on the Rayleigh–Plesset equation. The results in this work will help to determine or understand the main effect mechanisms of surface tension on acoustic cavitation and how the surface tension can help to improve the application efficiency.

## 2. Materials and Methods

### 2.1. Experimental Setup

**Figure 1:**
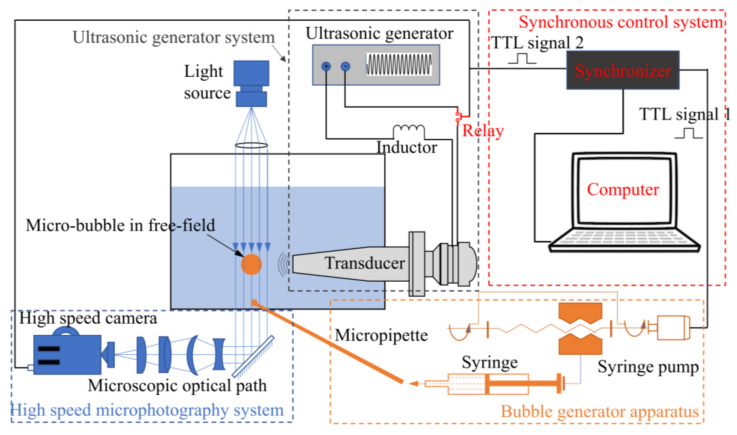
The schematic diagram of the experimental set-up.

The experimental setup in the present work mainly includes bubble generation apparatus, an ultrasonic generator, high-speed photography, and a synchronous control system, as shown in Figure 1. Single air bubbles were generated in a regulated co-flow micropipette injector described by Palanchon et al. [29]; the acoustic waves were generated by an acoustic transducer whose resonant frequency is 20.47 kHz (Institute of Acoustics, Chinese Academy of Science, China); an ultrafast high-speed camera (FASTCAM SA-Z, Photron, Japan) attached to an inverted fluorescence microscope (Axio observer 3, Zeiss, Germany) was used to image cavitation microbubbles in free-field at 480,000 fps; the synchronous control technique was applied in this work to adjust the start time of these three parts. More information about each part and how they work has been given in detail in Wu et al. [30].

**Figure 2:**
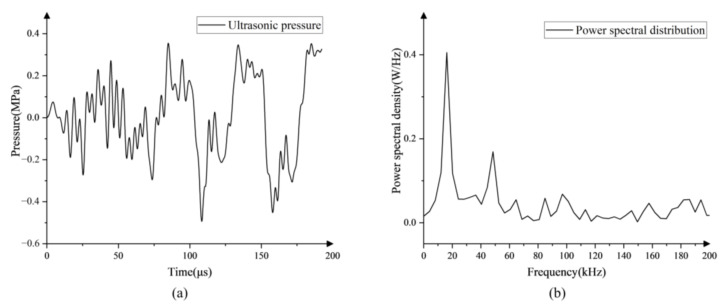
(**a**) Pressure-time profile measured in the liquid and (**b**) the corresponding frequency spectrum graph.

In the present work, the generated bubbles would rise in a line without any other boundaries around them under different flow speeds. The distance between two adjacent bubbles is relatively large (larger than 800 μm) compared to the bubble’s radius (15–40 μm), so the bubbles would not influence each other and can be regarded as in free-field; the acoustic pressure was measured with a hydrophone (TC4013, RESON, Denmark) positioned 1 cm in front of the transducer surface in the water where the targeted bubble caught and imaged, as shown in Figure 2; the temporal evolution of the bubble dynamics was recorded at 480,000 frames per second from the bottom view of the water tank with a resolution of 2.0 μm/pixel.

### 2.2. Materials

Sodium dodecyl sulfate (SDS, ≥99.0% (GC)) was purchased from Sigma-Aldrich. The solutions in the present work were all made using Milli-Q water with a conductivity of less than 10^−6^ S/cm at 21 °C. Pure deionized water and four different concentrations of SDS solutions were prepared for this work. A force tensiometer (K100, KRÜSS GmbH, Hamburg, Germany) and a viscometer (HAAKE™ Viscotester™ E, Thermo Scientific™, Waltham, MA, USA) were, respectively, used to measure the surface tension and the viscosity of the solutions. The temperature in the solutions was not controlled but monitored at around 21 °C. The properties of the solutions were measured over 8 times under the same conditions and then taken on average. As shown in Table 1, the measured results are listed for pure deionized water and SDS solutions with different concentrations. In order to discuss the results more easily, we defined these five kinds of liquid as Liquid A (pure deionized water), Liquid B (1.0 × 10^−5^ mol/L SDS aqueous solution), Liquid C (1.0 × 10^−4^ mol/L SDS aqueous solution), Liquid D (1.0 × 10^−3^ mol/L SDS aqueous solution), Liquid E (1.0 × 10^−2^ mol/L SDS aqueous solution) in order of decreasing surface tension value. As shown in Table 1, from Liquid A to E, the liquid surface tension decreases obviously, meanwhile, other liquid properties including the density and viscosity remain constant.

### 2.3. Image Analysis

All image analysis was done using Fiji (distribution of the ImageJ software, US National Institutes of Health, Bethesda, MD, USA). Data graphing was done using Origin (OriginLab, Northampton, MA, USA) and statistical analysis was performed using SPSS (IBM, Armonk, NY, USA).

Image sequences where individual bubbles were seen to generate and oscillate within the imaging field of view were used to extract the outline of the bubbles for further analysis. These images were cropped and enhanced to improve contrast. Some parts of the background were eliminated by removing objects smaller than 4 pixels using the Analyse particles plugin. Then, the outline of the bubble was then created using the ‘outline’ function in the binary menu of Fiji. These steps were completed for all time points during a bubble’s generation and movement. 

The x-y coordinate of the binary outline of the bubble was built for recording the bubble’s localization during exposure to the ultrasound. The origin coordinate was defined at the center of the bubble at time zero (*t* = 0) which was defined as the moment that the ultrasonic generator is triggered to work.

### 2.4. Analysis of Bubbles’ Typical Characteristics

The equivalent radius of a bubble at each time point was calculated and plotted as a function of time from the binary images of the single bubble. Circularity was assumed to calculate the equivalent radius (*R*) by *R* = √(A/π), where A is the bubble area. *V*_b_ represents the bubble’s volume. The assumption of spherical bubbles in this study is valid as the single bubble only oscillates in the imaging field of field. The center of the bubble was defined as centroid of the binary image of a single bubble by assuming a homogeneous distribution of the gas inside the bubble. The center of the bubble was used to represent the location of the bubble in this work

## 3. Results

As the fundamental unit of acoustic cavitation in most applications, a free-field single bubble plays a vital role. To assess the effect mechanism of the surface tension on acoustic cavitation, a single bubble in free-field exposed to ultrasound was investigated in liquids with different surface tensions. In addition, the R-P equations in the liquid with different surface tension were solved, in which the acoustic parameters are obtained from the measuring results of the ultrasonic field (Figure 2) by fifth harmonic extraction [31].

### 3.1. Time Evolution of Bubble Shape in the Free-Field

The temporary evolution of a free-field bubble in an ultrasonic field was recorded in pure deionized water (Liquid A, σ = 72.59 mN/m) and different concentrations of SDS aqueous solutions (Liquid B, σ = 58.75 mN/m; Liquid C, σ = 49.34 mN/m Liquid D, σ = 38.47 mN/m; Liquid E, σ = 27.13 mN/m). It was found that single bubbles in liquids behaved similarly, they oscillate and move in the same direction that the ultrasound wave propagated.

**Figure 3:**
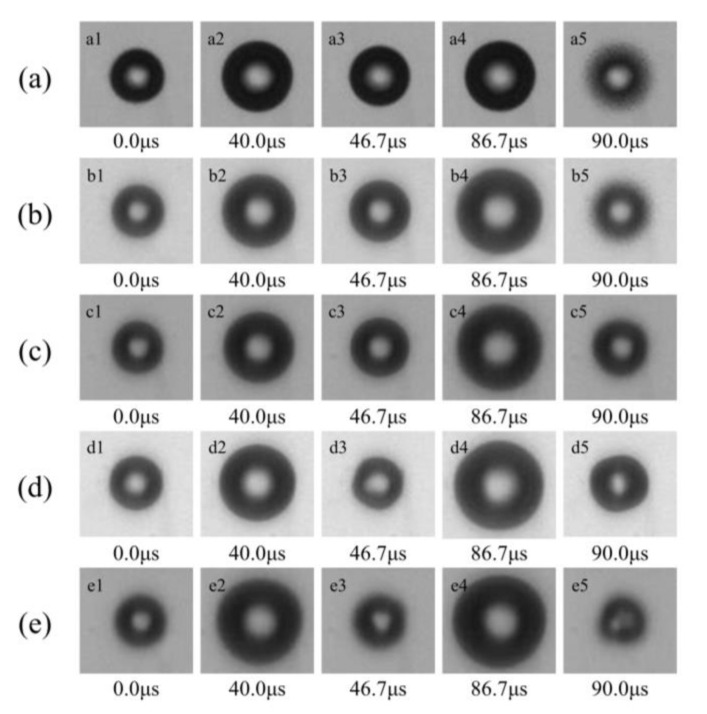
Temporal evolutions of the single bubble in free-field exposed to ultrasound in (**a**) Liquid A (surface tension *σ* = 72.59 mN/m); (**b**) Liquid B (surface tension *σ* = 58.75 mN/m); (**c**) Liquid C (surface tension *σ* = 49.34 mN/m); (**d**) Liquid D (surface tension *σ* = 38.47 mN/m); (**e**) Liquid E (surface tension *σ* = 27.13 mN/m). (*R*_0_ = 20 μm, T = 21 °C).

As shown in Figure 3, the typical outlines of the bubble are chosen in two cycles of the dominant frequency ultrasound. A single bubble is generated in free-field with an initial radius of 20 μm, as shown in Figure 3(a1–e1). Then, during two cycles of oscillating, the bubble expands to the maximum size of each cycle, as shown in Figure 3(a2–e2, a4–e4). It is found that the maximum radius during the bubble oscillating is obviously larger in the higher concentrations of SDS aqueous solutions. Meanwhile, the bubbles in the higher concentrations of SDS aqueous solutions deviate from the spherical shape after the bubble expands to its maximum size (Figure 3(d3,d5,e3,e5)). This might be the influence of the liquid’s surface tension, and the influence would be greater when the speed of the bubble margin is higher. In addition, it is also found that the bubble would move a little to the left (the red arrow in Figure 3) after several microseconds.

### 3.2. The Characteristics of Bubble Oscillate versus Time in Free-Field

In this section, the bubble radii and volume versus time in different liquids were analyzed. Meanwhile, the theoretical analyses were conducted by solving a modified R-P equation [32], which is shown in the following equation:

(1) $$R\left(\frac{d^{2}R}{dt^{2}}\right)+\frac{3}{2}{\left(\frac{dR}{dt}\right)}^{2}=\frac{1}{\rho }\left[\left(P_{0}+\frac{2\sigma }{R_{0}}\right){\left(\frac{R_{0}}{R}\right)}^{3\gamma }-P_{A}-P_{0}-\frac{2\sigma }{R}\right],$$

where *R* is the radius of the bubble, t is the time, ρ is the density of the liquid, σ is the surface tension coefficient, γ is the coefficient of heat insulation which in the present work is defined as 1.4, $P_{0}$ is the static pressure and $P_{A}$ is the acoustic pressure that has been measured by a hydrophone in the present study as shown in Figure 2.

**Figure 4:**
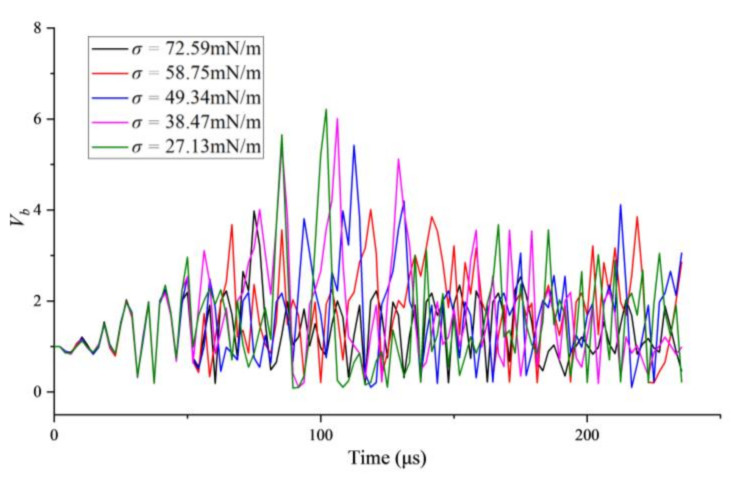
The volume of the single bubble versus time in liquids with different surface tensions.

**Figure 5:**
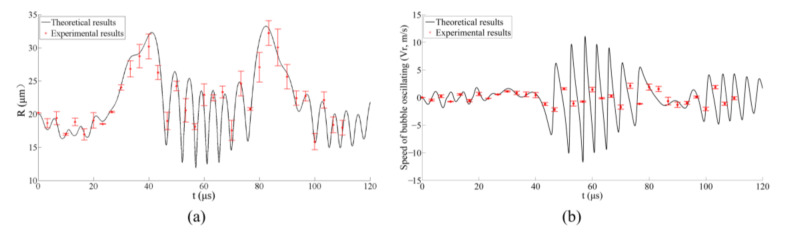
Comparison of the theoretical and experimental results for a free-field bubble in an ultrasonic field in the pure water (*R*_0_ = 20 μm); (**a**) results of the bubble radius and (**b**) the bubble oscillating speed.

The volume of the bubble (*V_b_*) versus time in liquids with different surface tension is shown in Figure 4, which can be found that the bubble in liquid with lower surface tension would oscillate fiercer with smaller volume in the peak phase of ultrasound and bigger volume in the trough of ultrasonic wave. The comparisons of the theoretical and experimental bubble radius in the water as a function of time are shown in Figure 5a. Figure 5b is the oscillating speed of the bubble margin which is defined as *dR*/*dt* and calculated based on the data in Figure 5a. The red asterisks and the black line indicate the experimental and the theoretical results, respectively. It is notable that the theoretical model shows agreement with the measured results of the bubble’s radius except for the speed of the bubble oscillating. Due to the relatively long shooting interval of a high-speed camera, the experimental results of bubbles’ speed are much different from the theoretical results when the speed changes dramatically, but they are consistent in trend, as shown in Figure 5b.

**Figure 6:**
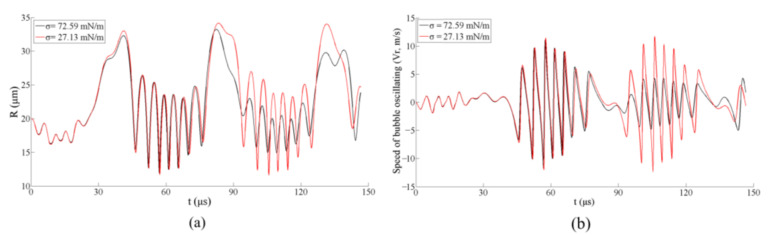
Comparison of the theoretical results for a free-field bubble in the pure water and 1.0 × 10^−2^ mol/L SDS aqueous solution (*R*_0_ = 20 μm). (**a**) Results of the bubble radius and (**b**) the bubble oscillating speed.

In order to further investigate the relationship between the liquid’s surface tension and the bubble motions in the free-field, the theoretical results of a single bubble in water (*σ* = 72.59 mN/m) and SDS aqueous solution (*σ* = 27.13 mN/m) are compared, as shown in Figure 6. It is found that the maximum radius of the bubble in the SDS aqueous solution is larger than that in the water, especially during the second cycle of oscillating (during 50–100 μs), as shown in Figure 6a, which is consistent with the experimental results shown in Figure 3 and Figure 4. In addition, the bubbles’ oscillating speeds are also much different during the second cycle, the bubble in the SDS aqueous solution oscillates much more fiercely (Figure 6b), which might significantly affect the stability of the bubble (Figure 3(d3,d5,e3,e5)). As a result, the bubbles oscillating in liquid would experience reduced resistance when the surfactant is added, which leads to more violent motions and larger bubble sizes. This also might be the reason for the dissymmetric bubble shape in the liquid with relatively low surface tension. As the speed of the bubble motion increases when the surface tension of the liquid decreases, the bubble is more susceptible to asymmetric force field, (e.g., ultrasonic traveling waves and gravity) to form a non-spherical shape (Figure 3(d3,e3,d5,e5)).

**Figure 7:**
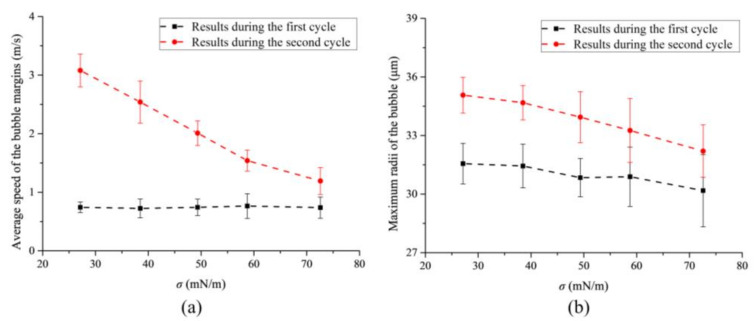
(**a**) The average speed and (**b**) the maximum bubble radius during bubble oscillating as functions of the liquid’s surface tension.

Furthermore, the bubbles’ maximum radii and the average speeds of the bubble margin during the first and second cycles are analyzed based on the bubbles’ temporary evolutions in the liquid with different surface tensions. As shown in Figure 7, the average speed (Figure 7a) and the maximum radius (Figure 7b) of the bubble both show a decreasing trend as the surface tension of the liquid increases, but the changing trends during the second cycle are much more obvious. As the bubbles oscillate more violently during the second cycle (Figure 3, Figure 6b and Figure 7a), it can be concluded that the influence of surface tension on the free-field single bubble might be closely associated with the bubble’s oscillating speed. In other words, the fiercer the bubble motion, the greater the effect of the liquid’s surface tension on the bubble’s dynamics. This finding can also be used to explain why the differences in the bubble shape in the liquid with different surface tensions are much more obvious during and after the bubble’s collapse, as described in our previous work.

### 3.3. The Displacement of the Bubble versus Time in Free-Field

**Figure 8:**
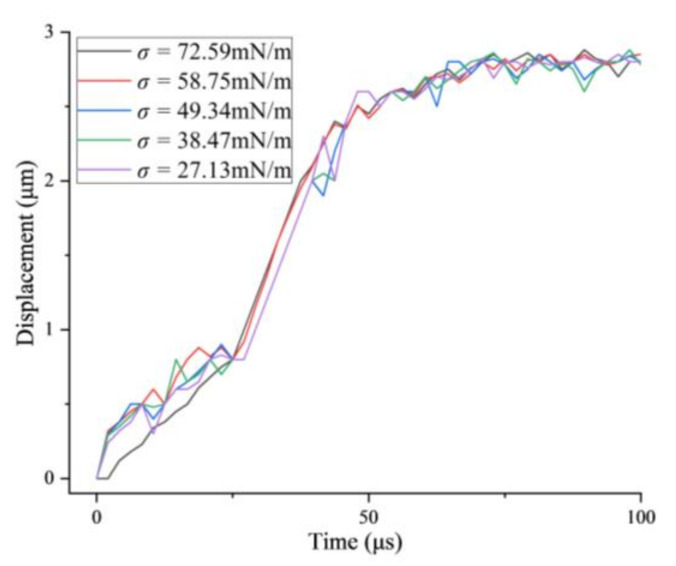
The average displacement of the single bubble versus time in liquids with different surface tensions.

It had obviously been found that there was a displacement of the bubble during its oscillation in different liquids which was resulting from the transmission of the ultrasound. In different liquids, the driving force from the traveling ultrasound can be regarded as equal as the intensity of the ultrasound and the density of the liquid were constant in all the experiments. In the present work, the position of the bubble’s center was analyzed by image sequences of the bubble, and the relative position versus time, which was defined as the distance to the origin at each time point, was calculated in liquids with different surface tensions, as shown in Figure 8. Different colors represent the relative position of the bubble in different liquids (positive value represents the same direction as the ultrasonic transmission), which have a similar variation tendency. From these results, it is found that the surface tension has little influence on the displacement of a single bubble in an ultrasonic field in the present work, which reflects that the viscosity of the liquid might be the main reason that influences the bubble’s movement.

## 4. Discussion

The effects of the liquid medium’s surface tension on the dynamics of the single free-field bubble in an ultrasonic field are investigated in the present work. Synchronous high-speed microscopic imaging is proposed to record the bubble evolutions for investigating the bubble outline. For bubbles in the free-field, the combined theoretical and experimental method is used to research the effects of the liquid’s surface tension on the bubble dynamics, which include the maximum radius, oscillating speed, and the destabilization of the bubble. The related findings are as follows:

1. As the surface tension of the liquid decreases, the maximum radius, oscillating speed, and the destabilization of the bubble increase when it oscillates in each cycle.
2. As time goes on, the maximum radius, oscillating speed, and the destabilization of the bubble increase during the later oscillating cycle, then the bubble dynamics are found to be more sensitive to the surface tension of the liquid.

In summary, the surface tension of liquid mediums significantly changes the bubble dynamics in an ultrasonic field. The lower the surface tension of the liquid is, the more intense the dynamics of the bubble in the free-field are. In addition, how much the bubble motion would be affected by the liquid’s surface tension is based on the intensity of the bubble motion. These results give an insight into the influence mechanism of the surface tension on the single acoustic cavitation bubble and would provide more information for the use of the surfactants in the applications of acoustic cavitation to facilitate their optimizations.

Based on these findings, the influence of surface tension on a bubble’s motion has been partly revealed. As we all know, the surface tension on the bubble’s boundary is highly related to the bubble’s radius, so it is also vital for us to perform further research on this aspect to provide more foundations for revealing the effect mechanisms of surface tension on the bubble’s motion in an ultrasonic field.

## Figures and Tables

**Table 1:** Physical characteristics of liquid media.

Name	Content	Concentration (c, mol/L)	Density ^1^ (*σ*, g/cm^3^)	Surface Tension ^1^ (*σ*, mN/m)	Viscosity ^1^ (*μ_l_*, cP)
Liquid A	Deionized water	—	0.99	72.59	1.00
Liquid B	SDS aqueous solution	1.0 × 10^−5^	0.99	58.75	1.00
Liquid C	SDS aqueous solution	1.0 × 10^−4^	0.99	49.34	1.00
Liquid D	SDS aqueous solution	1.0 × 10^−3^	0.99	38.47	1.00
Liquid E	SDS aqueous solution	1.0 × 10^−2^	0.99	27.13	1.00

^1^ Values of surface tension, density, and viscosity of the liquid samples at 25 °C.
